# B-flow imaging in abdominal ultrasound: a pictorial essay

**DOI:** 10.1007/s40477-025-01068-x

**Published:** 2025-08-21

**Authors:** Marco Becciolini, Alice Brighenti, Valeria Tiraferri, Antonio Corvino, Andrea Boccatonda, Carla Serra

**Affiliations:** 1 Misericordia di Pistoia, Pistoia, Italy; 2 Scuola SIUMB, Pisa, Italy; 3 Diagnostic and Therapeutic Interventional Ultrasound Unit, IRCCS Azienda Ospedaliero-Universitaria di Bologna, Policlinico Sant’Orsola-Malpighi, via Massarenti n 9, 40138 Bologna, Italy; 4 Medical, Movement and Wellbeing Sciences Department, University of Naples “Parthenope”, via Medina 40, 80133 Naples, Italy

**Keywords:** Abdomen, B-Flow, Doppler, Vascular, Ultrasound

## Abstract

B-Flow technology represents a significant advancement in vascular ultrasound imaging, offering high-resolution visualization of blood flow without the limitations inherent in conventional Doppler techniques. By employing Coded Excitation and Tissue-Blood Equalization (TBE) technologies, B-Flow enhances the detection of low-velocity and microvascular blood flow while minimizing aliasing artifacts and angle dependency. This innovative technique proves especially useful in abdominal ultrasound, enabling the accurate assessment of vascularization in organs such as the liver, kidneys, pancreas, spleen, and bowel. B-Flow’s ability to depict fine vascular details is particularly beneficial in evaluating focal lesions, vascular malformations, portal hypertension, and transplant perfusion. Furthermore, the technique’s application extends to both parenchymal and vascular pathologies, demonstrating significant advantages over traditional Doppler methods in terms of spatial resolution and real-time flow visualization. This pictorial essay aims to describe the technological basis of the B-Flow technique, highlighting its advantages and disadvantages, as well as the main clinical applications in abdominal diagnostics.

## Introduction

Ultrasound is a widely used imaging modality to evaluate the abdomen due to its safety, non-invasiveness, and accessibility. Traditionally, the characterization of intra-abdominal vascularization is performed using color-Doppler techniques, which, however, have some limitations, such as dependence on the insonation angle and the presence of aliasing artifacts. B-Flow technology (GE HealthCare) represents a significant innovation in vascular ultrasound, allowing for detailed visualization of blood flow without the use of Doppler by directly representing information related to moving blood [1, 2]. B-Flow is a flow mode that images blood reflectors and tissue information simultaneously, providing an accurate morphological display of the intraluminal blood flow throughout the entire field of view [1, 2].

## Physical and technological principles of B-flow

B-Flow utilizes Coded Excitation technology to enhance weak blood flow signals. Unlike Color Doppler and Power Doppler, which visualize flow based on the frequency shift caused by the Doppler effect, B-Flow employs advanced digital processing to amplify the signal from blood cells while simultaneously suppressing signals from surrounding tissues [3]. Coded ultrasound pulses are transmitted with long waveforms that contain a significant amount of energy. During reception, the digital beamformer decodes the long echo waveforms into sharp and crisp short pulses. This process enables deep penetration and tight resolution simultaneously.

B-Flow also uses a Tissue-Blood Equalization (TBE) technology to suppress tissue signal. The Coded Excitation technology boosts both blood flow signal and tissue signal. The TBE technology can differentiate the flow signal from tissue and apply more amplification to the flow signal and less amplification on the tissue [1, 2].

This technology allows for high-resolution grayscale images similar to those of conventional B-mode imaging, but with improved visualization of vascularization. Furthermore, thanks to its high spatial resolution and its ability to reduce Doppler artifacts, B-Flow is particularly useful in studying microvascularization and small-caliber vessels [1, 2].

This pictorial assay employs the GE Healthcare implementation of B-flow, which integrates proprietary speckle-reduction and adaptive filtering algorithms. While the underlying B-flow principle is vendor-agnostic, performance may vary across platforms.

## B-flow applications

B-Flow can display the small vessel flow signals in gray scale throughout the entire field of view. This non-Doppler technique uses Coded Excitation to capture the hemodynamics within large vessels and perfusion of smaller vessels through organs [4]. B-Flow is not angle-dependent and visualizes real flow without overwriting of vessels. Finally, the user can choose to visualize B-Mode and B-Flow in a dual screen or use Hybrid Visualization, which shows the B-Flow overlaid on the B-Mode image. The dual-screen modality is useful because it makes it possible to do measurements (or select an area) in the B-mode and show it on the B-Flow image and vice versa.

B-Flow may better display vessel diameter, high spatial resolution to show fine vessel details and flow hemodynamics in larger vessels, and no angle dependency or ROI needed, absence of aliasing artifacts [5]. Moreover, the technique is characterized by advanced digital processing allows for the visualization of vascular details with a higher resolution compared to color-Doppler. B-Flow is particularly effective in visualizing vessels with low flow velocities, such as hepatic and renal vessels [1].

## Clinical applications in abdominal ultrasound

The use of the B-Flow technique is spreading in abdominal imaging for the evaluation of the vascularization of various organs and vascular districts. The main applications include parenchymal and vascular diseases.

### Liver

**Fig. 1:**
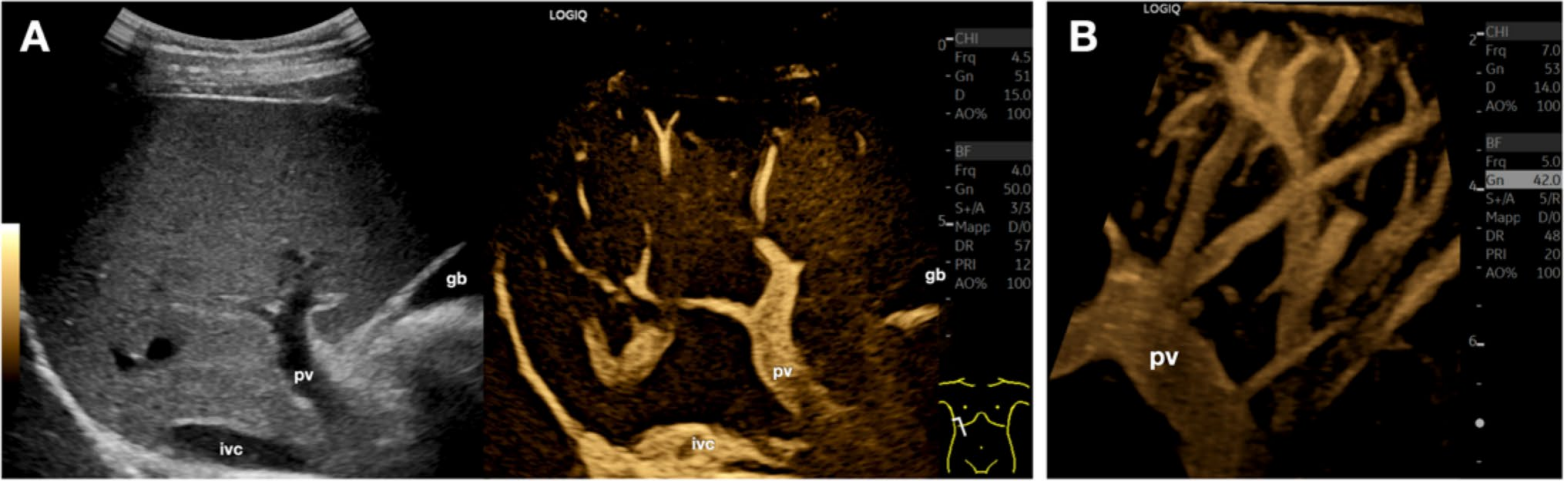
Normal liver anatomy with detailed visualization of the vascular system. In **A**, B-Flow dual-screen intercostal sonogram of the liver in a healthy subject demonstrates a fine depiction of the vascular anatomy. **B**. A more detailed image of the liver vascular anatomy can be achieved with B-Flow Cine Capture by acquiring a clip while tilting the transducer in the intercostal space. *Gb* indicates the gallbladder; *ivc*, inferior vena cava; *pv*, portal vein

B-Flow has proven particularly useful in evaluating the liver, allowing detailed visualization of intrahepatic vascularization, especially of the portal vein and its branches (Fig. 1) [2]. This technique is particularly valuable for defining specific morphological and anatomical details and assessing the patency of the vascular systems.

**Fig. 2:**
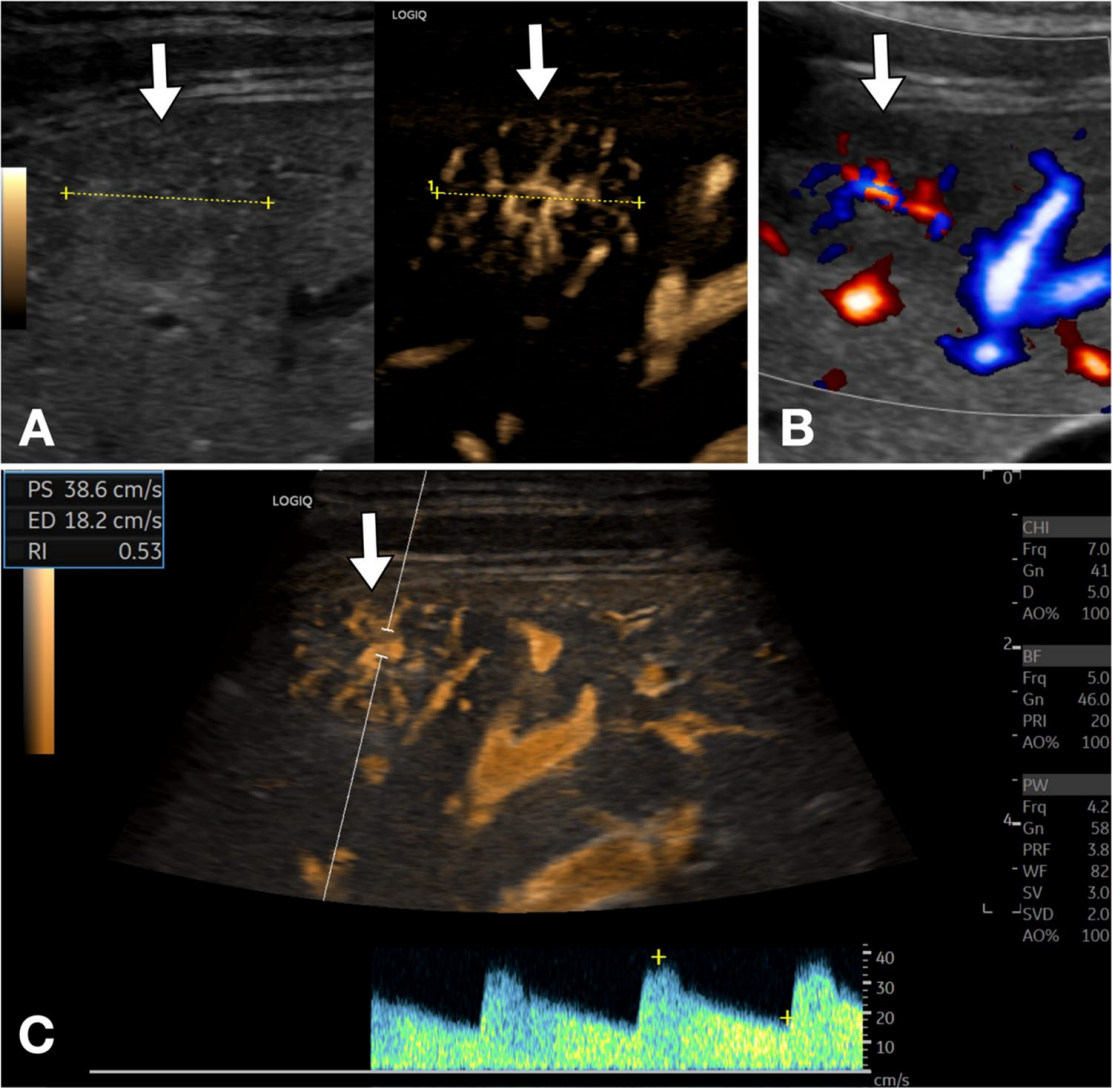
Liver FNH. **A**. Dual screen B-Flow sonogram demonstrates a heterogeneous, slightly hypoechoic liver lesion in the subcapsular area. Note that B-Flow enables better visualization of the characteristic “spoke wheel” appearance, with vascularization supported by a central arteriole, as well as a clear definition of its size (approximately 2 cm, between calipers). **B**. For comparison, directional power Doppler also shows internal vascularization, but fails to show exactly the “spoke wheel” feature. **C**. Additionally, B-Flow allows for improved identification of a vessel suitable for perform Doppler sampling, showing a low resistance index (0.53)

**Fig. 3:**
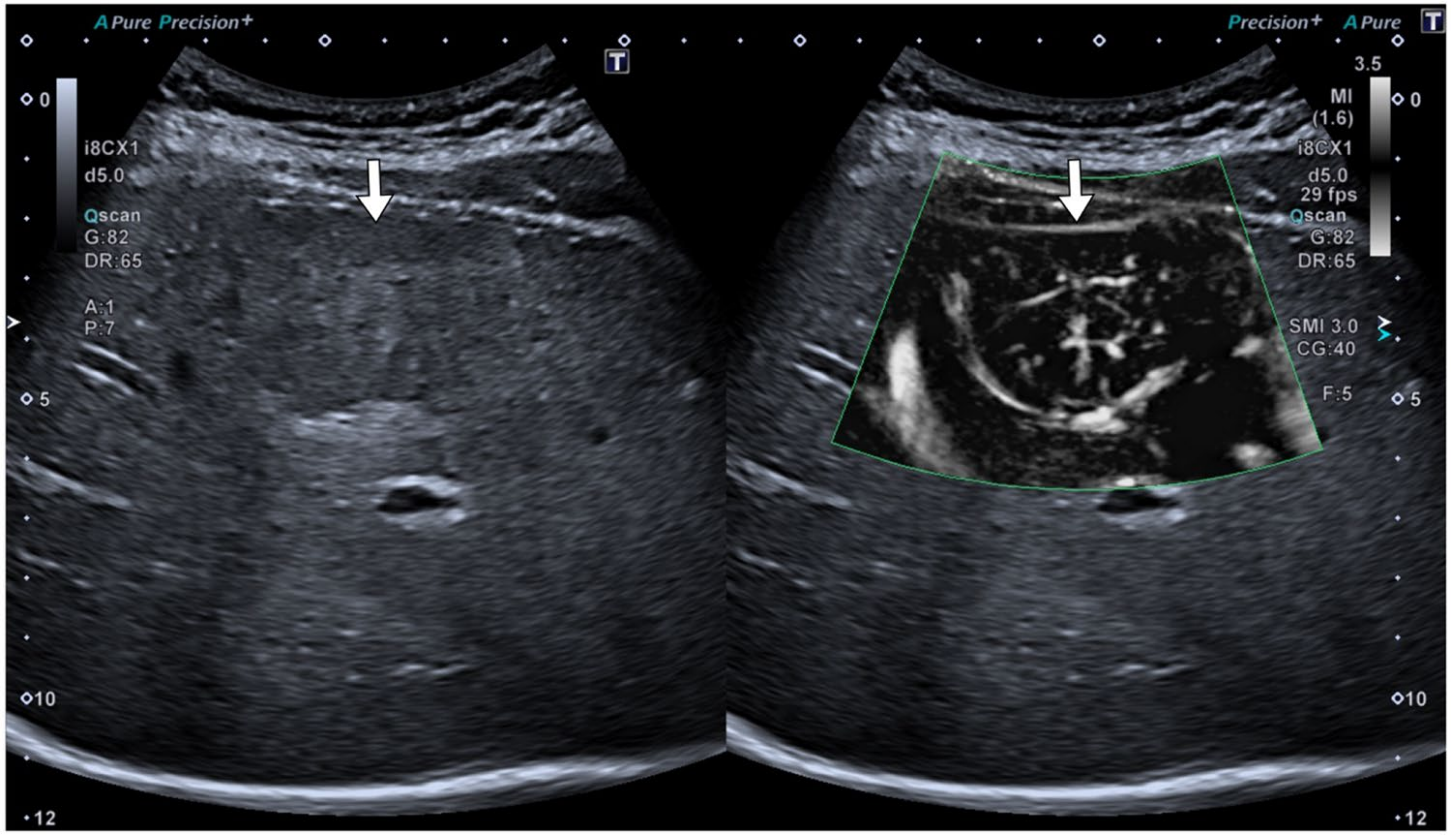
Liver focal nodular hyperplasia (FNH). Superb Microvascular Imaging (Canon Medical). Dual screen sonogram demonstrates a heterogeneous, slightly hypoechoic liver lesion in the subcapsular area. Superb Microvascular Imaging (SMI) also allows visualization of the “spoke wheel” appearance, consistent with FNH

**Fig. 4:**
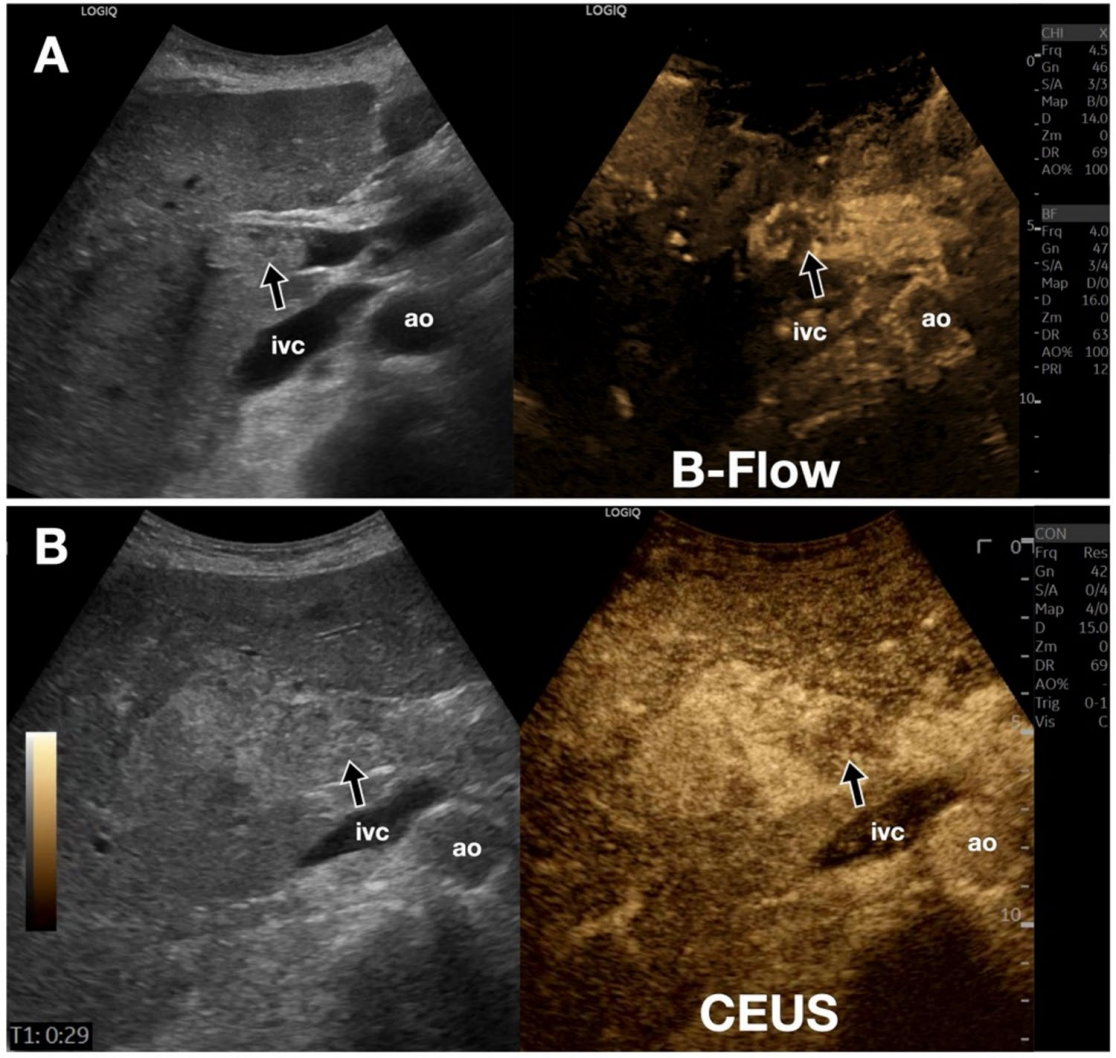
Liver. Portal vein thrombosis. **A, B**. Case of occlusive thrombosis of the main trunk of the portal vein. In **A**, B-Flow sonogram at the level of the porta hepatis. In **B**, comparison with a CEUS image. Vascularization of the portal vein thrombus (black arrow) is clearly visualized with B-Flow and confirmed with CEUS. The thrombus appears perfused, suggesting a neoplastic nature of the thrombosis. *Ao* indicates the abdominal aorta; *ivc*, inferior vena cava

Regarding liver diseases, focal liver lesions such as hepatocellular carcinoma, adenomas, and metastases can be characterized by their vascularization with greater detail than with conventional Doppler [6–8]. The lesion that exhibits the most peculiar pattern is undoubtedly focal nodular hyperplasia (FNH); it is characterized by a typical “spoke wheel” vascularization supported by a central arteriole, with low-resistance Doppler flow (Fig. 2, 3) [9]. Moreover, B-Flow is useful in the detection of splanchnic vein thrombosis, thus enabling a clearer distinction between the thrombus and residual blood flow compared to color-Doppler [10]. Moreover, B-flow can allow identification of the nature of the thrombus (Fig. 4) by detecting internal vascularization in malignant thrombosis. Therefore, B-Flow allows an accurate analysis of portal flows, useful for evaluating portosystemic shunts and esophageal varices. Indeed, the analysis of the spleno-portal venous system is fundamentally important in patients with chronic liver diseases. B-Flow is particularly useful in detecting reversed or reduced flows, allowing for the early diagnosis of portal hypertension [11].

### Kidneys and urinary tract

**Fig. 5:**
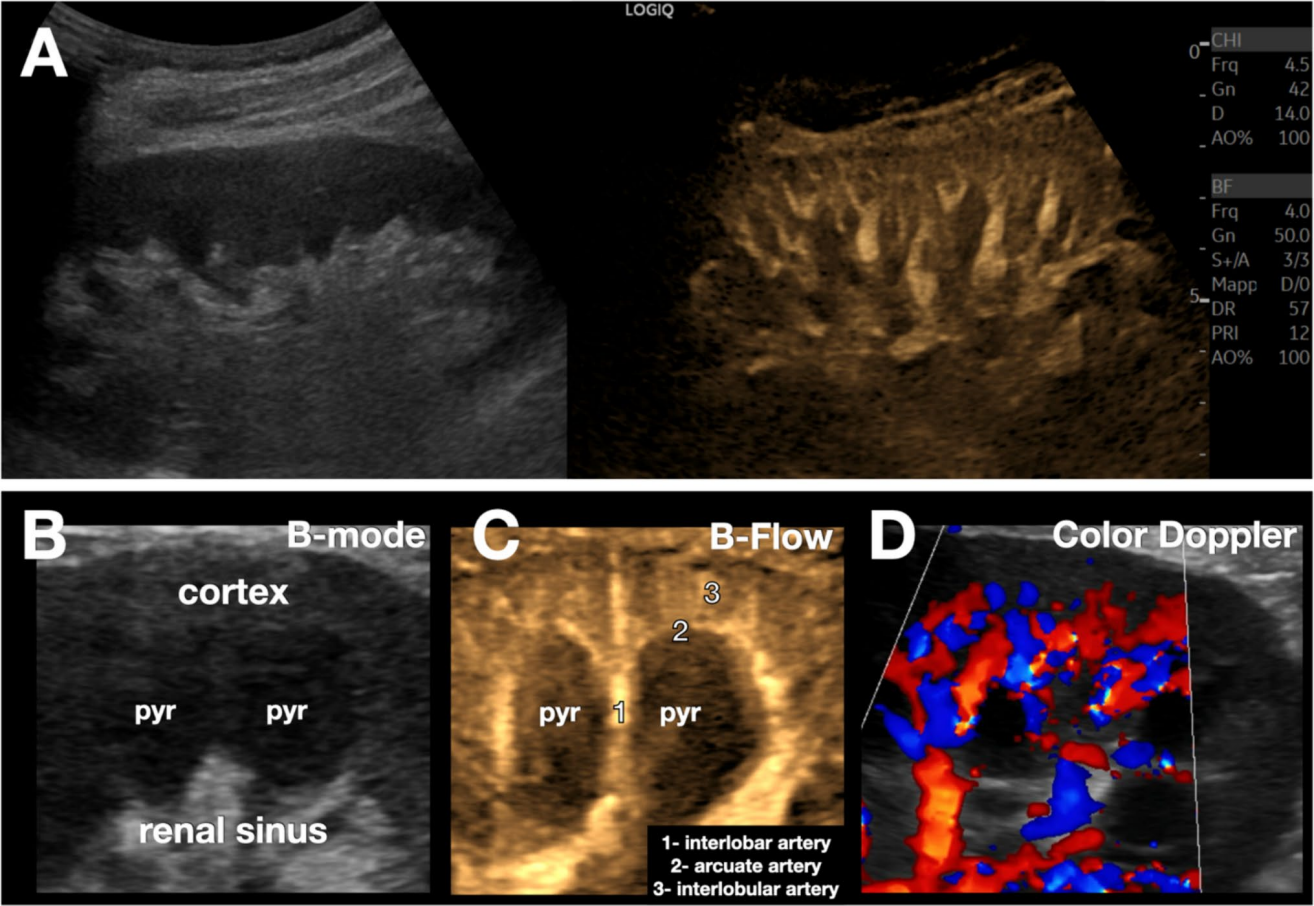
Normal kidney vascular anatomy. **A**. B-Flow allows for enhanced detail of intraparenchymal vascularization with a convex transducer (C1-6). **B-D**. Magnified image of the kidney obtained with an L2-9 transducer, showing on the left (**B**, B-mode) the normal hypoechoic pyramid (pyr), the hyperechoic renal sinus, as well as the cortex. In the center (**C**), B-Flow clearly depicts the interlobar artery (1), arciform (2), and the thin interlobular arteries (3). On the right (**D**), a color Doppler image is provided for anatomical comparison: the B-Flow image shows finer vessel anatomy

**Fig. 6:**
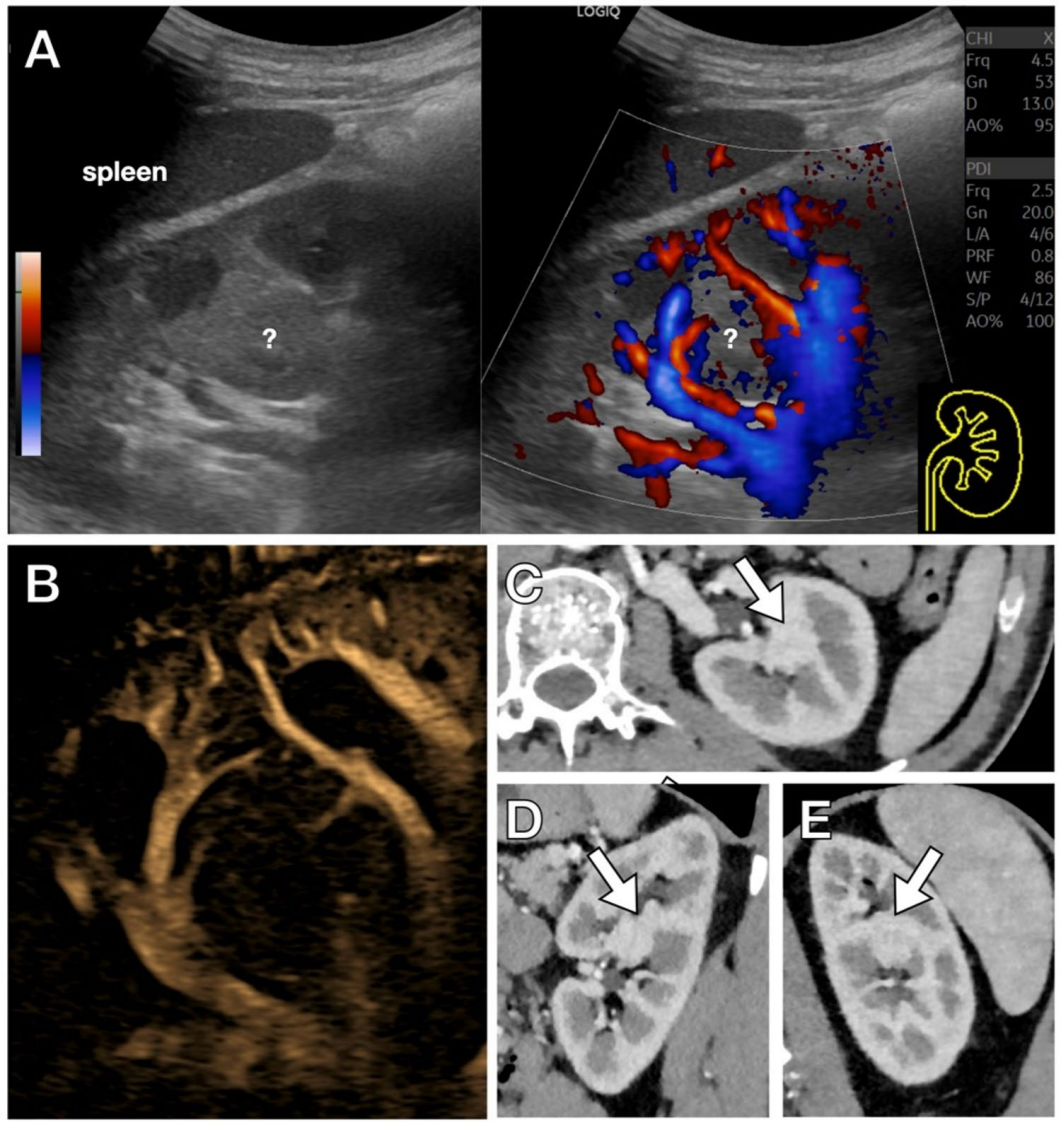
Kidney anatomical variation. **A**. Dual-screen directional power Doppler sonogram obtained with a convex probe. B-mode ultrasound shows a pseudo-nodular solid image (indicated by “?”) in the left kidney. By using power Doppler with a low PRF, the vascularization of the area seems regular, but noise artifacts are present. **B.** Using a higher frequency transducer (L2-9) with B-Flow, the vascular detail is even more clearly depicted, thereby excluding aberrant vascularization. **C–E**. Axial (**C**), Sagittal (**D**) and Coronal (**E**) contrast-enhanced multiplanar CT reconstruction in the arterial phase, previously performed due to tumor suspicion, confirms the presence of a hypertrophied Column of Bertin (white arrow)

**Fig. 7:**
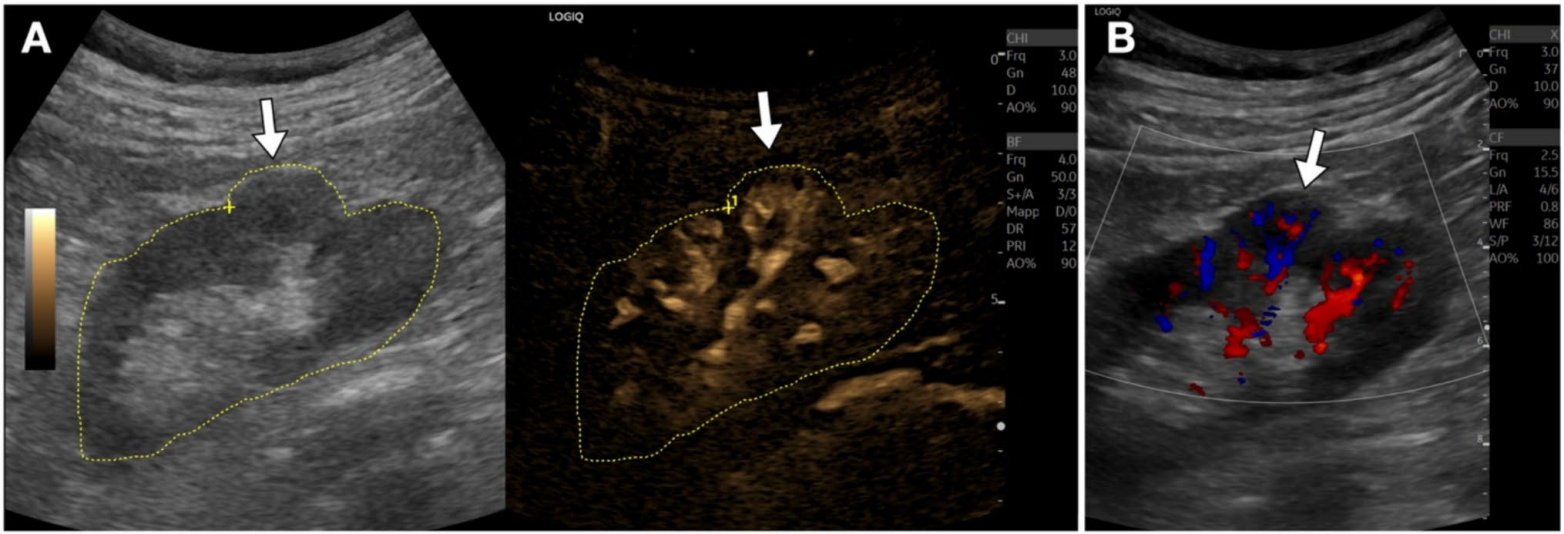
Kidney anatomical variation. **A**. On B-mode ultrasound, a cortical bulging is observed in the mid-third of the renal cortex. This appearance may raise suspicion of an atypical formation (tumoral lesion). The B-Flow detail demonstrates regular vascularization in this area, continuous with that of the adjacent parenchyma, supporting the diagnosis of a normal anatomical variant. **B**. Color Doppler sonogram, for comparison. The diagnosis of a normal variation can already be achieved with this technique; however, B-Flow allows a better depiction of the normal renal vasculature

B-Flow provides detailed visualization of the kidney’s normal vascular architecture (Fig. 5), helping to avoid misdiagnoses of normal variations, such as the hypertrophied Bertin column (Fig. 6) and dromedary hump (Fig. 7) which may be mistaken for masses, particularly by less experienced operators [12].

**Fig. 8:**
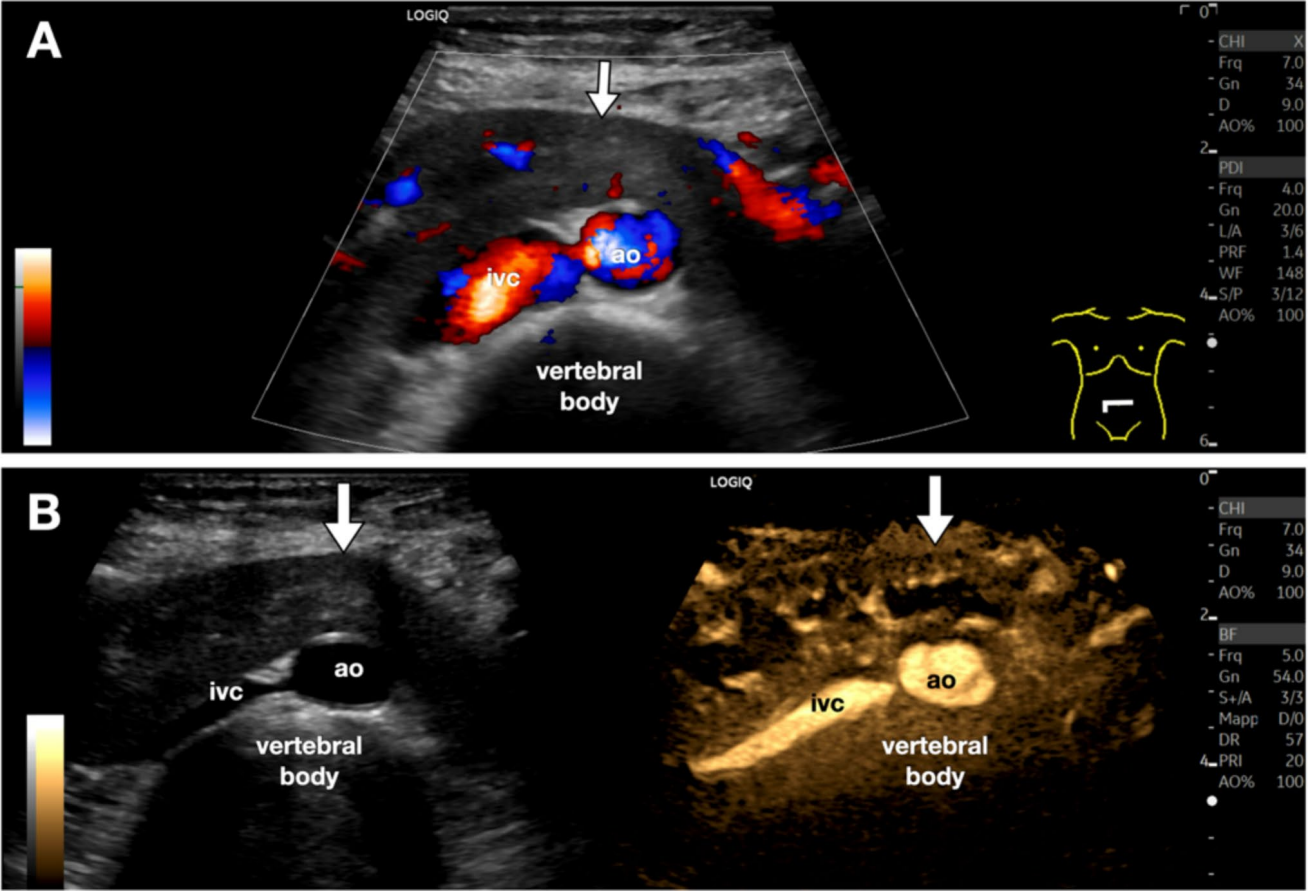
Horseshoe kidney. Variant. **A**. Axial directional power Doppler sonogram showing a parenchymal bridge (white arrow) anterior to the abdominal aorta (ao) and inferior vena cava (ivc), with internal vascular flow. **B**. B-Flow imaging provides a clearer depiction of the normal renal vascularization, confirming the diagnosis

Another rare variation is the so-called horseshoe kidney, with a prevalence of around 1/400 births [13]. The diagnosis is already obtained by demonstrating the connection between the two lower poles of the kidneys with a parenchymatous “bridge” superficial to the abdominal aorta, however, B-Flow may enhance the examiner’s confidence by clearly depicting the normal renal vascularization (Fig. 8).

In the context of vascular nephropathy and renal transplantation, B-Flow is useful for evaluating renal perfusion and for the identification of renal artery stenosis, providing a better definition of blood flow compared to conventional Doppler [14–17]. In particular, the technique can better define any morphological anomalies or stenoses affecting the renal venous or arterial systems. This can be useful in patients being evaluated for renovascular hypertension or in the follow-up of anastomoses in post-renal transplant patients.

**Fig. 9:**
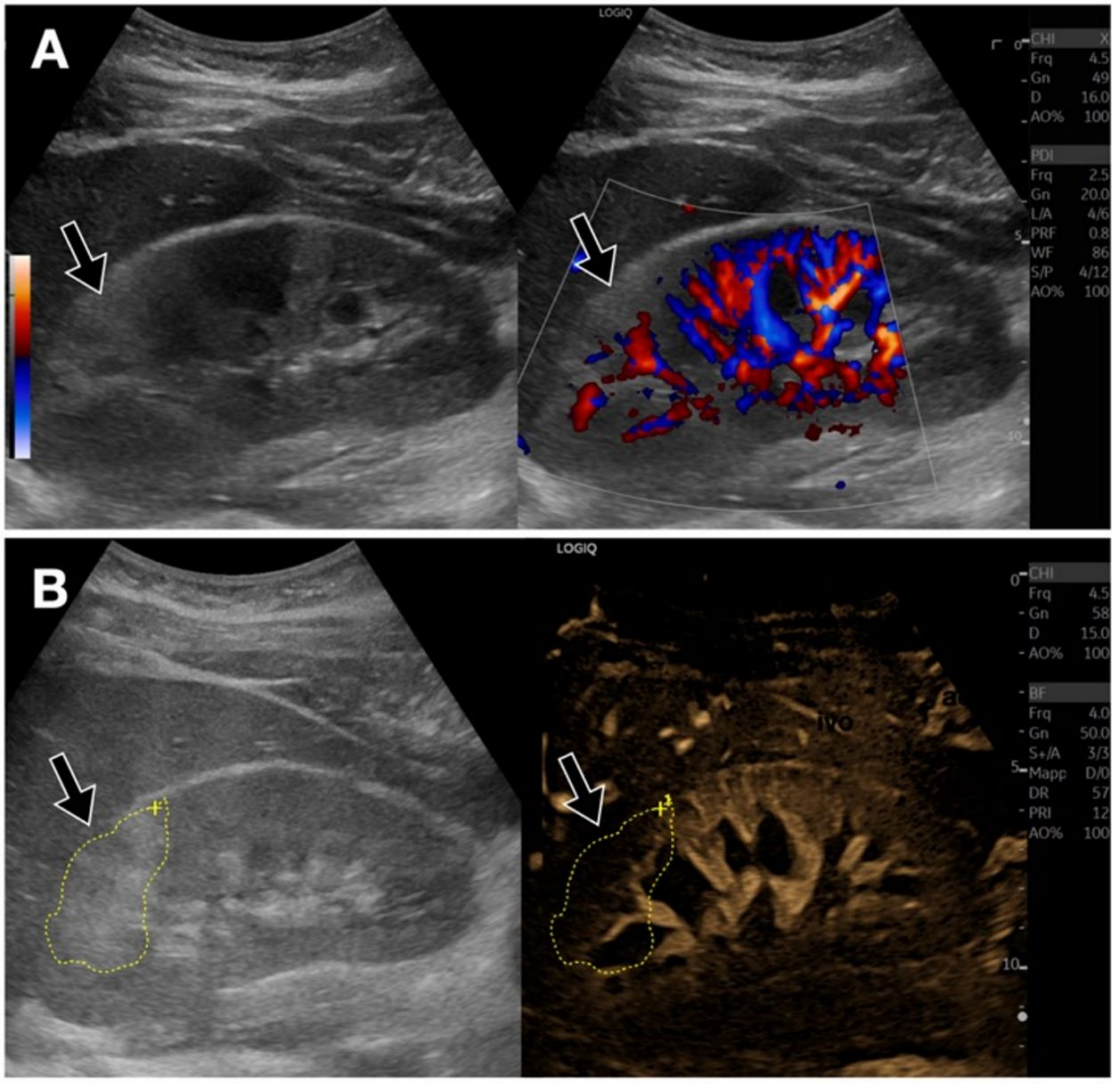
Acute pyelonephritis. **A-B**. Dual-screen power Doppler (**A**) and B-Flow (**B**) sonogram in a patient with fever and a diagnosis of acute pyelonephritis. On B-mode, an area of hyper-echoic heterogeneity is observed in the upper third of the kidney. Power Doppler already detects an alteration in kidney perfusion, which is more evident when using B-Flow (black arrow, encircled area)

Acute pyelonephritis is a topic of growing interest [18]. The visualization of an area with altered cortico-medullary perfusion, in an appropriate clinical context, allows for the diagnosis of acute pyelonephritis (Fig. 9). In this setting, a completely avascular focal area should suggest a possible evolution towards abscess formation [18, 19]. Renal infarctions, which often present with a triangular morphology, are included in the differential diagnosis.

**Fig. 10:**
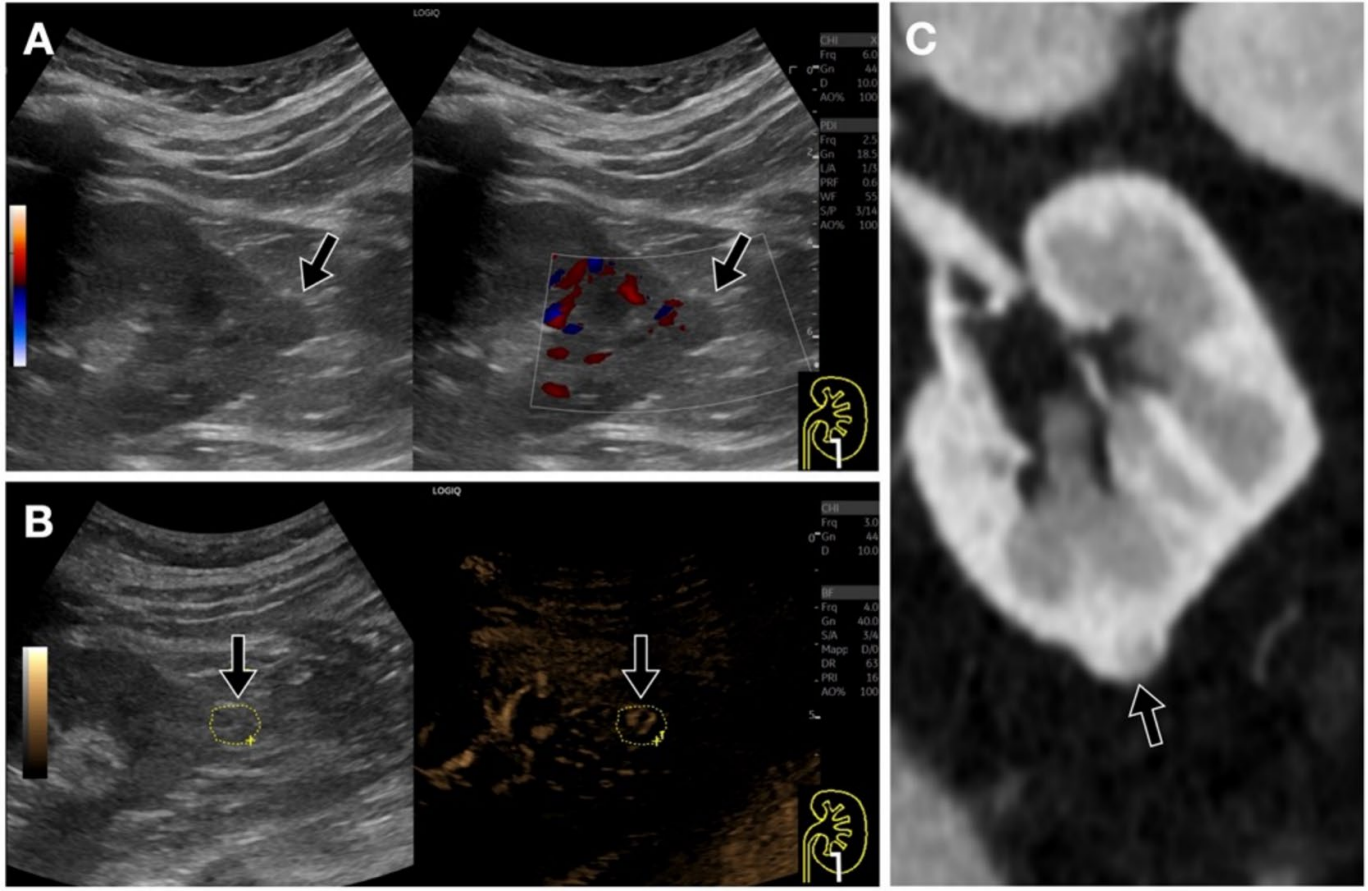
Small renal tumor. **A**. Dual-screen power Doppler sonogram showing a small, suspect nodule (black arrow) at the lower pole of the left kidney, with no clear vascularization. **B**. The B-Flow technique, compared with conventional Doppler, enables a more detailed visualization of aberrant vascularization within the mass, differentiating it from a complex cystic lesion or normal cortical bulging. The area is encircled in the B-mode image to help locate it in the B-Flow sonogram. **C**. Coronal contrast-enhanced multiplanar CT reconstruction confirms the presence of a solid lesion. Histopathology confirmed a clear cell carcinoma (TNM 1a)

**Fig. 11:**
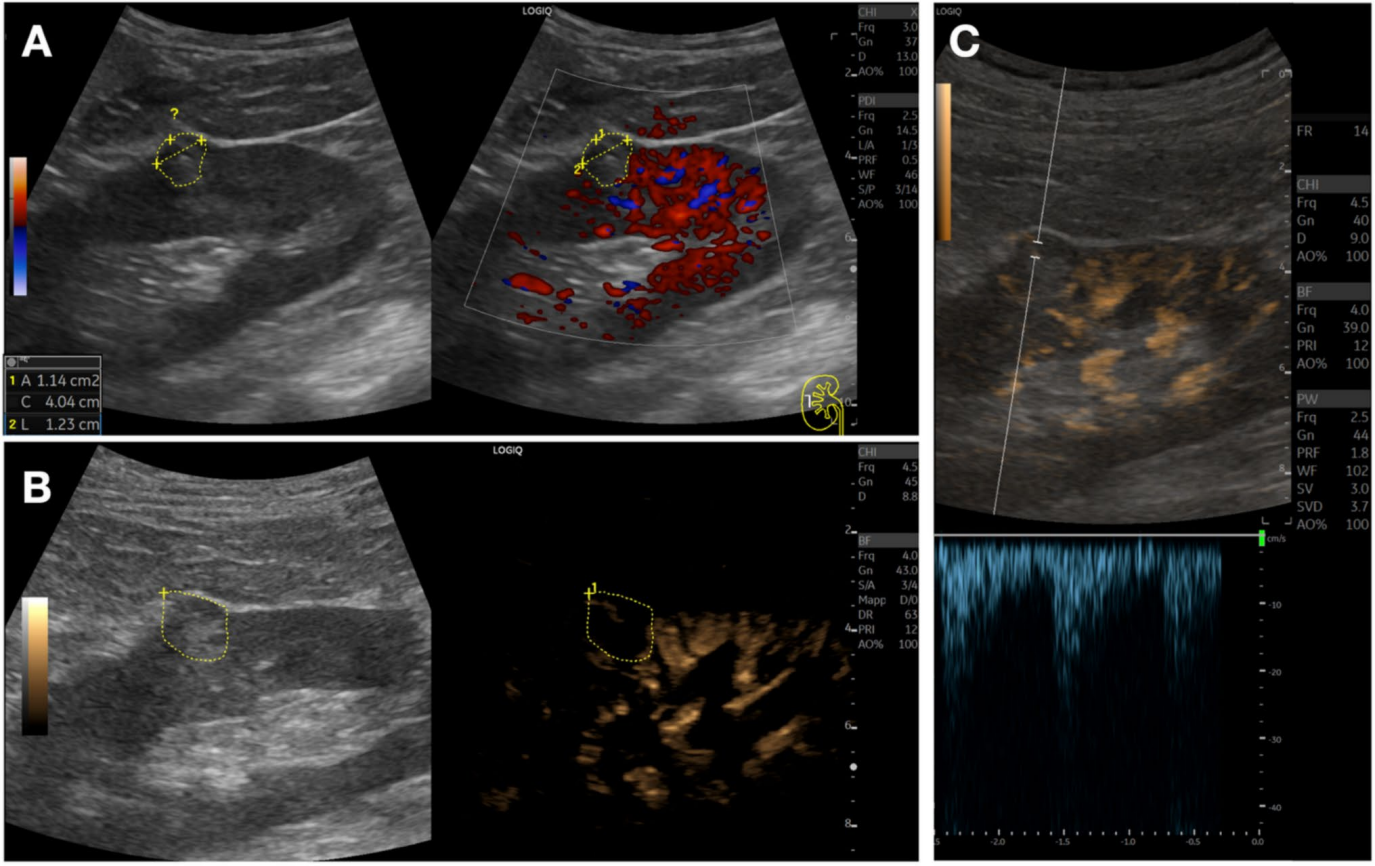
Small renal tumor. **A**. Dual-screen power Doppler sonogram showing a centimetric hyperechoic nodule (encircled area, ?) at the middle third of the left kidney, with no clear vascularization. **B**. The B-Flow technique, compared with conventional Doppler, enables the visualization of a small intralesional (encircled are) vessel. **C**. B-Flow enables Doppler sampling, confirming an arterial spectrum. This was also a clear cell carcinoma (histopathological diagnosis)

**Fig. 12:**
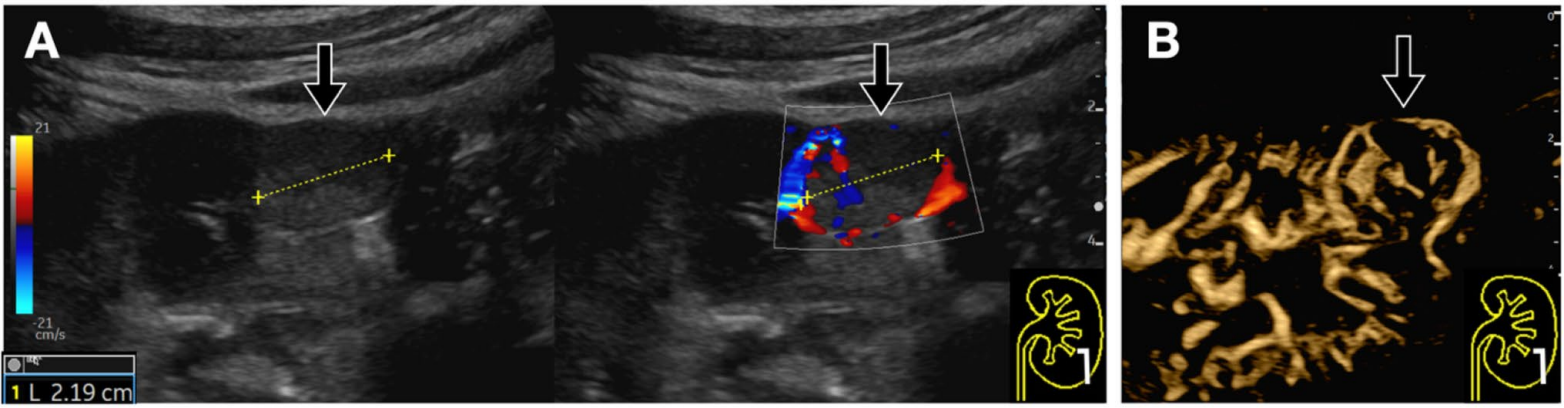
Renal tumor (oncocytoma). **A**. Dual-screen power Doppler sonogram showing a slightly hyperechoic suspect nodule (black arrow) in the lower pole of the left kidney, mainly with peripheral vascularization. **B**. The B-Flow technique, compared with conventional Doppler, enables a more detailed visualization of a “spoke wheel” vascularization pattern of the mass, suggesting a possible oncocytoma, confirmed after surgery

Regarding focal kidney lesions, although there are no specific patterns, b-flow may help to differentiate solid lesions from non-anechoic cysts (e.g. with proteinaceous content), by showing internal vascularization, which may be less evident with conventional color or power Doppler, in particular for small tumors (Figs. 10, 11, 12).

**Fig. 13:**
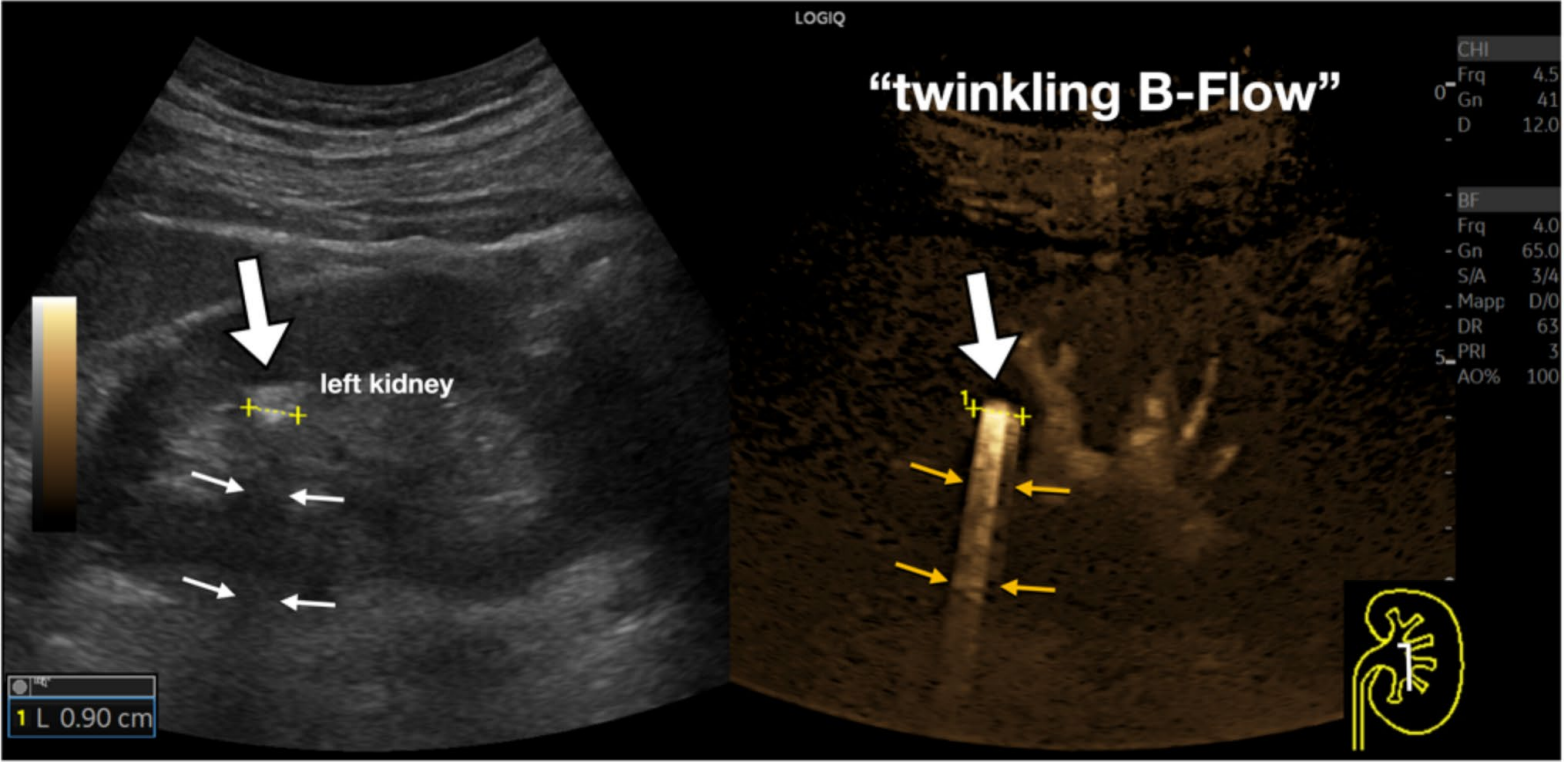
Kidney stone. On B-mode, a small, hyperechoic focus (9 mm) (white arrowhead) can be demonstrated in the upper third of the left kidney, with a subtle posterior shadow (small arrows). The B-Flow technique accentuates the appearance of an artifact posterior to the calcific formation ("twinkling B-Flow sign"). Note that, differently from other images provided in this pictorial, the Pulse Repetition Interval (PRI) is lowered to 3 to enhance the artifact while suppressing the normal kidney vasculature

Moreover, B-Flow may give useful details for studying the urinary tract. Twinkling artifact is a well-known artifact that improves the detection of kidney stones. This artifact can be visualized with B-flow as well (Fig. 13). As a technical consideration, the Pulse Repetition Interval (PRI) should be lowered to avoid showing small vessels so that the twinkling artifact is more obvious.

**Fig. 14:**
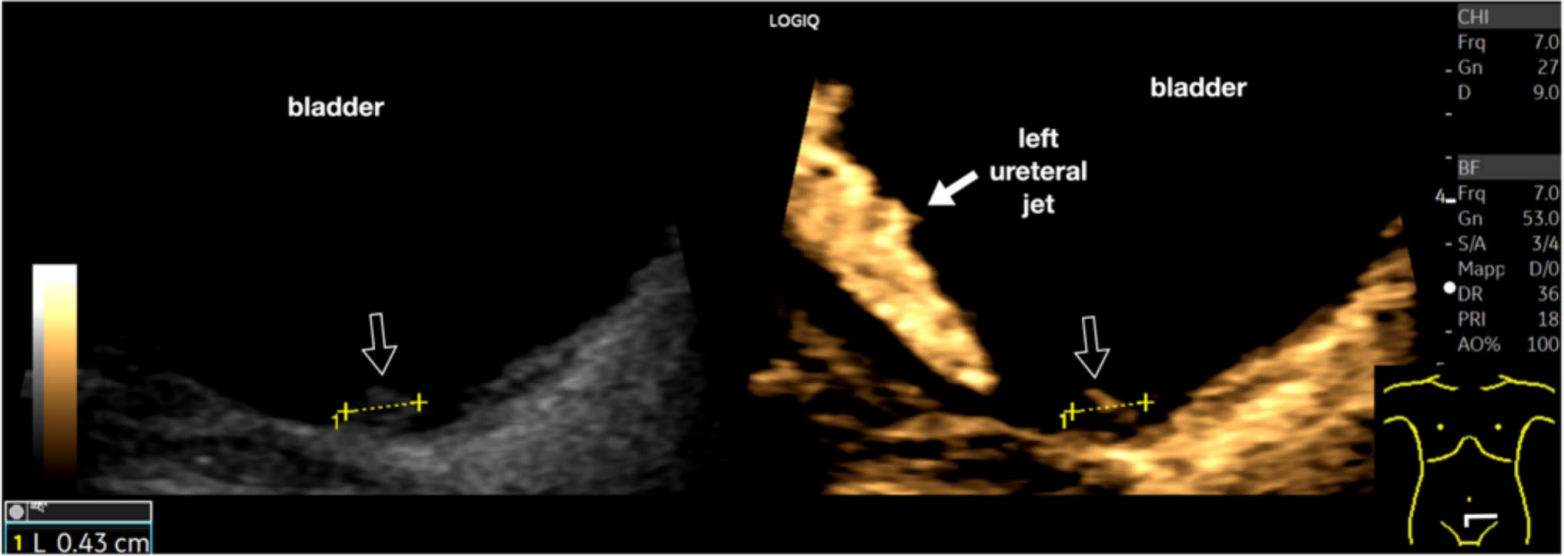
Small bladder tumor. Integrated B-Mode and B-Flow study of the bladder. The image provides two relevant findings. First, it demonstrates the physiological presence of the left ureteral jet (poorly appreciated in B-mode). Second, it reveals a small vessel inside a solid formation on the bladder floor, barely detectable with conventional B-mode ultrasound (between calipers, indicated by the black arrow), thereby facilitating a more accurate detection (urothelial formation confirmed on cystoscopy)

In the evaluation of the bladder, B-Flow can be used to show the normal ureteral jet and is. Particularly useful to demonstrate internal vascularization in small vegetating lesions (Fig. 14).

### Abdominal vascular diseases

**Fig. 15:**
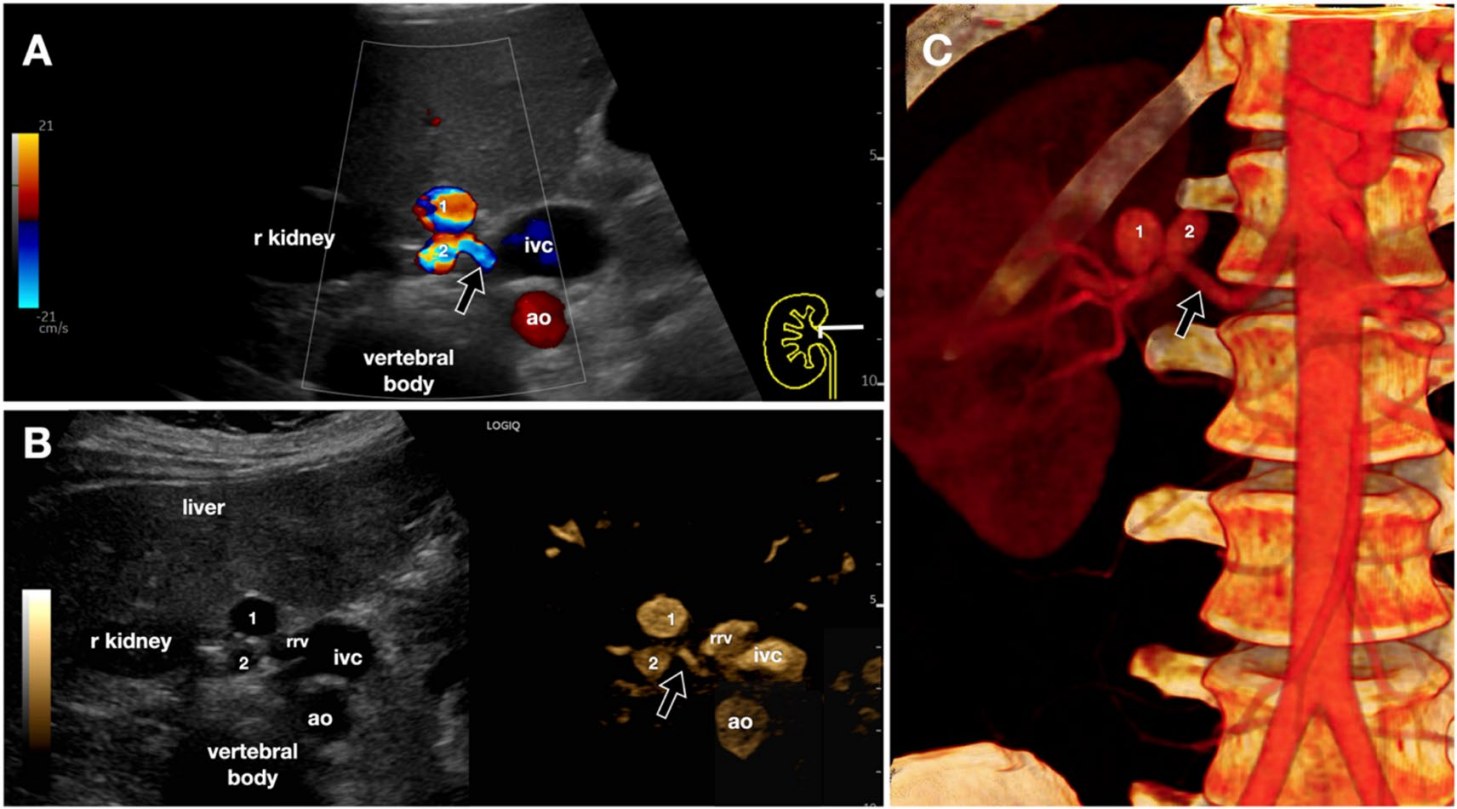
Patient with double renal artery aneurysms. **A-C**. Comparison between color Doppler (A), dual-screen ultrasound (B-mode and B-Flow, in **B**), and CT with volume-rendering reconstruction images (**C**). The figure demonstrates a good morphological and dimensional correlation between the aneurysms (1, anterior, bigger; and 2, posterior) of the right renal artery (black arrow) as visualized on B-Flow and on CT. *Ao* indicates the abdominal aorta; *ivc*, inferior vena cava; *r* kidney, right kidney; *rrv*, right renal vein

B-Flow can also be applied in the study of vascular pathologies of the abdominal aorta and visceral arteries (Fig. 15) [20]. It could be useful in the identification of stenoses and aneurysms, although it lacks some relevant data compared to color-Doppler, such as aliasing. Regarding dissection, while B-flow can depict the intimal flap and false lumen, contrast‐enhanced ultrasound (CEUS) remains the reference standard for endoleak detection, and B-flow studies have missed some endoleaks compared with CEUS [21, 22].

### Gallbladder, biliary tract and bowel diseases

**Fig. 16:**
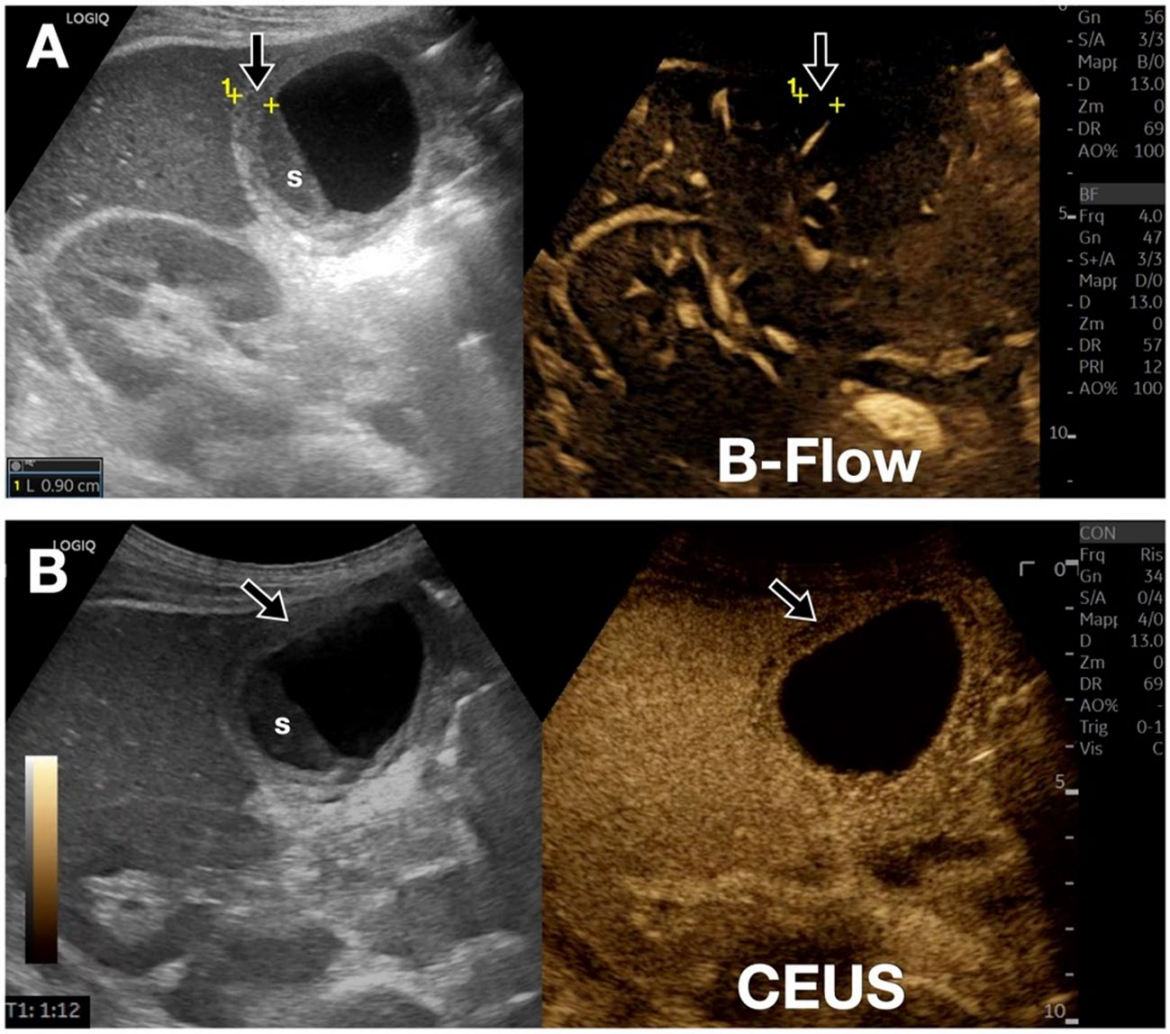
Patient with acute cholecystitis and echogenic intraluminal material. A-B. In **A**, the B-Flow technique, similar to CEUS (**B**), allows for optimal delineation of the hyperemic gallbladder walls (black arrow). Additionally, the absence of vascular signals within the echogenic material (s) inside the gallbladder excludes the presence of wall neoplasms

**Fig. 17:**
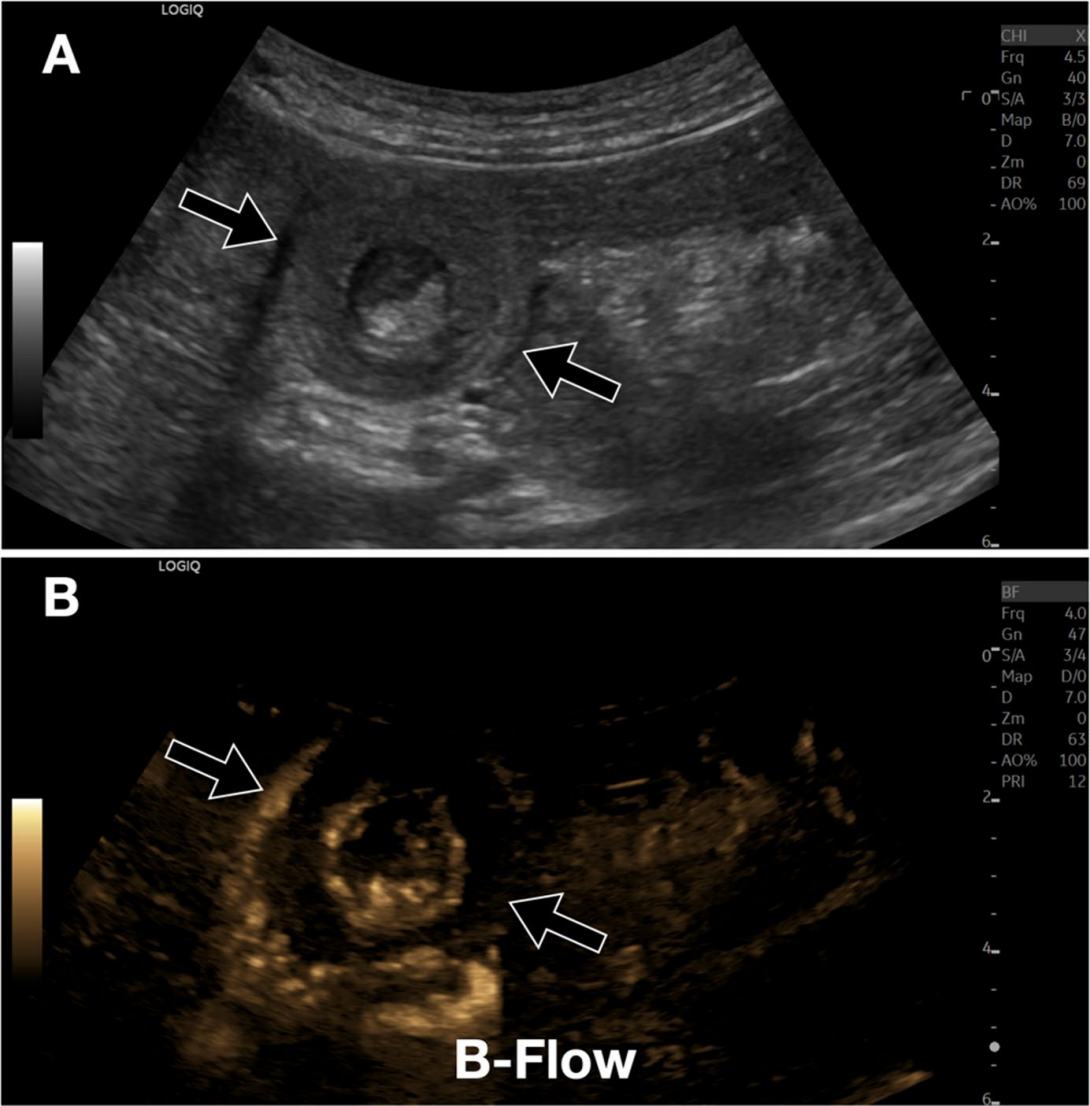
Intestinal volvulus. **A-B**. A patient presenting with an acute abdomen and ultrasound findings suggestive of intestinal volvulus. The B-Mode image (**A**), with its characteristic “whirlpool sign” appearance, raises diagnostic suspicion. The B-Flow technique (**B**) provides additional information on the degree of bowel wall vascularization, demonstrating hypoperfusion as a sign of ischemic compromise of the involved loop

The B-Flow technique can have several applications in the study of the gallbladder, bile ducts, and intestinal loops. In particular, regarding the gallbladder, the technique can optimize the evaluation of the wall and its margins (Fig. 16), as well as assess the vascularization of any polyps and/or wall masses [23]. In the context of biliary pathology, B-Flow can improve the visualization of intraluminal structures such as neoplastic masses (cholangiocarcinoma) [24]. Concerning intestinal loop pathologies, B-flow allows for an improved assessment of wall thickening and its vascularization (see Limberg score) [25]. Furthermore, the technique can provide important information in cases of suspected intestinal ischemia by evaluating the degree of wall perfusion (Fig. 17), even during follow-up after therapy.

### Lymph nodes

**Fig. 18:**
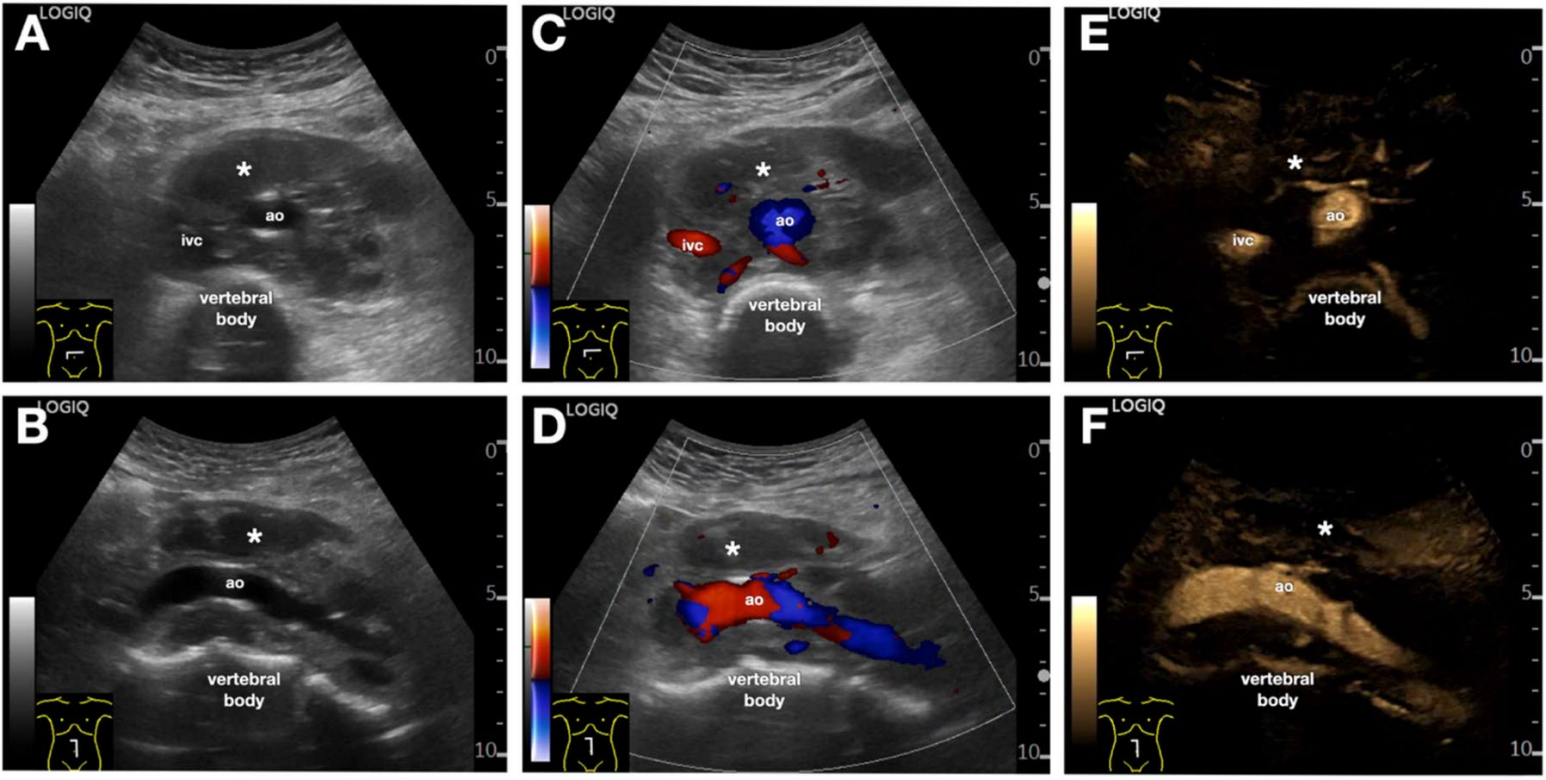
Retroperitoneal mass. **A–F** B-Mode (**A**, **B**),power Doppler (**C**, **D**) and B-Flow (**E**, **F**). The upper images are axial, whereas the lower sagittal. The images show a heterogeneous mass (*) that envelops the abdominal aorta (ao) and the inferior vena cava (ivc). The B-Flow allows for betterdefinition of the course of the vessels present within the mass with respect to power Doppler, therefore, it becomes an usefultool in guiding a biopsy procedure on the mass

Ultrasound examinations are often requested for abdominal masses (Fig. 18) or for evaluating lymph node stations in patients with suspected hematological disorders. Once one or more lymph nodes are identified, it is important to establish a presumptive diagnosis regarding their nature [26].

**Fig. 19:**
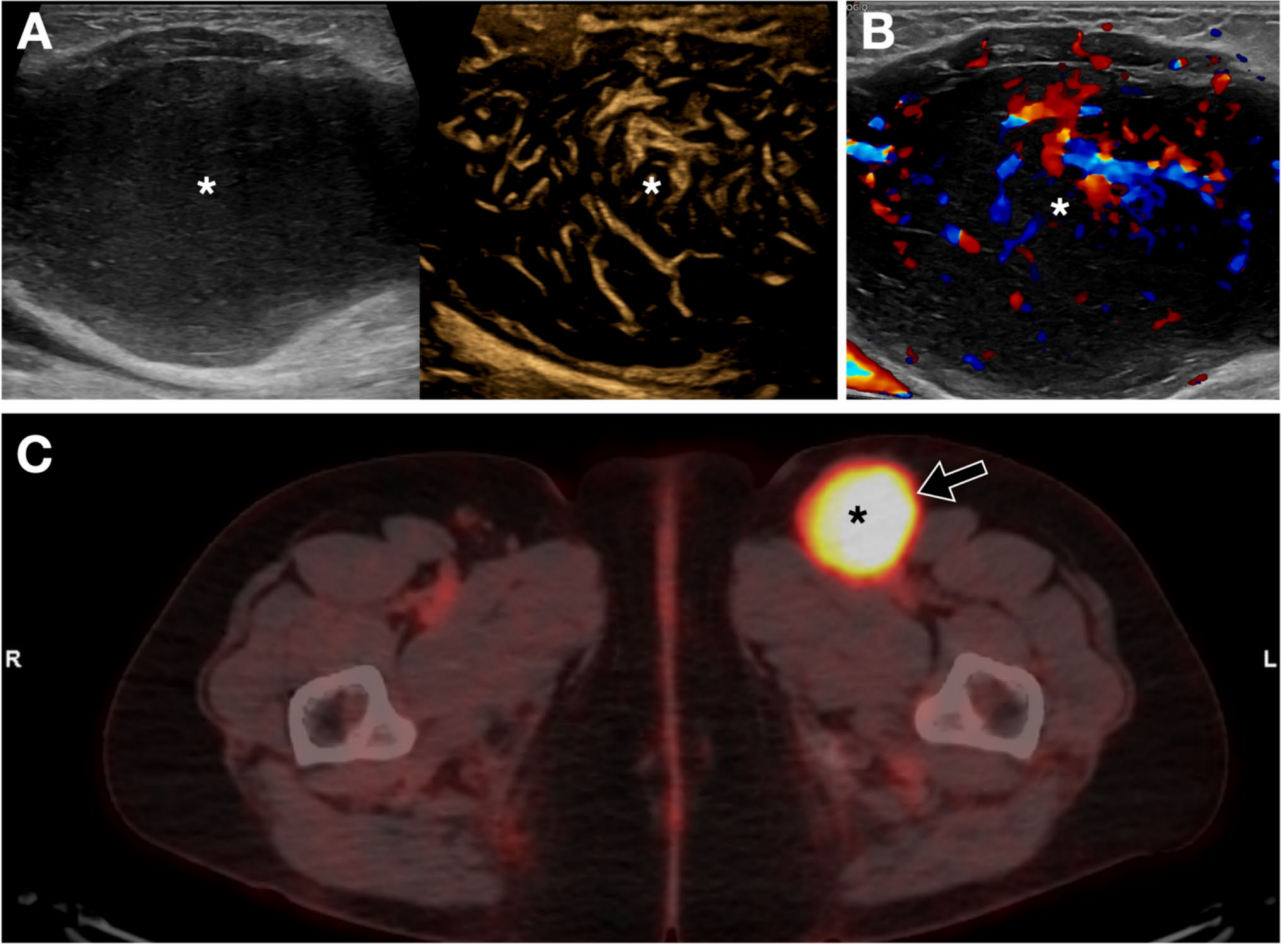
Pathological inguinal lymph node. **A**. An ultrasound evaluation of the abdomen and lymph node stations was requested in a patient with an inguinal mass. The examination was normal except for the left inguinal region, where a round, hypoechoic lymph node lacking a normal hilum was depicted (asterisk). The detailed vascular study performed with the B-Flow technique revealed aberrant vascularization, which, along with the B-mode characteristics, are features suggestive of malignancy in lymph node. **B**. Color Doppler comparative sonogram. The atypical vascularization is already clear with this modality. However, the details of the vessels are rougher to the B-Flow image. **C**. Correlation with axial PET-CT images shows tracer uptake by the lymph node on the left. The final diagnosis was grade 2 non-Hodgkin lymphoma, diffuse variant

The vascularization of the lymph node is one of the main differential criteria: homogeneous vascularization from a single vascular hilum is a sign of benignity, while disorganized and multifocal vascularization without a hilum is indicative of malignancy. The B-Flow technique can enable a more specific evaluation of these details (Fig. 19).

## Limitations of the B-flow technique and other microvascular techniques

Despite its numerous advantages, B-Flow presents some limitations. Although effective in visualizing micro-vessels, B-Flow can be less sensitive than Power Doppler in detecting extremely slow flows. Moreover, unlike Spectral Doppler, B-Flow does not allow for a quantitative evaluation of flow velocity. B-Flow may suffer from artifacts due to its sensitivity to flow, such as flash artifacts due to sudden tissue motion [27]. Eventually, the efficacy of the technique is influenced by the resolution of the ultrasound system and the quality of the signal obtained from the patient.

It should be noted that other ultrasound microvascular techniques exist, that can give finer vascular anatomical details with respect to conventional Color Doppler, similarly to B-Flow [28].

## Conclusions

B-Flow technique represents a significant innovation in abdominal ultrasound, enabling detailed visualization of blood flow without the limitations of conventional Doppler. Thanks to its high spatial resolution and its ability to reduce artifacts, it finds numerous applications in the evaluation of hepatic, renal, spleno-portal vascularization, and abdominal vascular pathologies. However, its use must be integrated with other imaging modalities to achieve a complete and accurate diagnosis.

## Data Availability

Not applicable.
